# Assessing the Behavioral and Personality Changes in Alcohol Dependence Syndrome in Wardha, Central India

**DOI:** 10.7759/cureus.48419

**Published:** 2023-11-07

**Authors:** Anaiska Ray, Sudhir Ninave, Pradeep S Patil, Sanjot Ninave, Tooba Khan

**Affiliations:** 1 College of Medicine, Jawaharlal Nehru Medical College, Datta Meghe Institute of Higher Education and Research, Wardha, IND; 2 Department of Forensic Medicine and Toxicology, Jawaharlal Nehru Medical College, Datta Meghe Institute of Higher Education and Research, Wardha, IND; 3 Department of Psychiatry, Jawaharlal Nehru Medical College, Datta Meghe Institute of Higher Education and Research, Wardha, IND; 4 Department of Anaesthesiology, Jawaharlal Nehru Medical College, Datta Meghe Institute of Higher Education and Research, Wardha, IND

**Keywords:** alcoholism, road traffic accident, alcohol dependence syndrome, alcohol addiction, alcohol dependence

## Abstract

Background

Alcohol dependence syndrome occurs when the consumption of alcohol is uncontrollable. Most of the alcohol drinkers are usually males. There is a rise in the incidence of road traffic accidents under the influence of alcohol due to locomotor and cerebral dysfunction. Alcohol is a significant cause and contributing factor for domestic violence, family disharmony, and displeasure in families. Research studies have shown that after the lockdown of COVID-19, the consumption of alcohol decreased in India. This study was conducted to assess the behavioral and personality changes in alcohol dependence syndrome.

Methods

This study was conducted at a rural tertiary care hospital in Wardha, Maharashtra, Central India. Sixty-two males participated in the study. Out of which, 56 were included in the study. There were urban and rural participants in the study. The study was conducted for a period of six months. The participants who were being treated for alcohol withdrawal and alcohol dependence syndrome were included in the study. The individuals unwilling to participate in the research and those admitted to the intensive care unit were excluded from this study. The primary outcome measure of the study was to assess the behavioral and personality changes in alcohol dependence syndrome. Participants were screened using the Cut-Down, Annoyed, Guilty, and Eye-Opener (CAGE) and the Alcohol Use Disorders Identification Test (AUDIT) questionnaires. The diagnosis of alcohol dependence syndrome was made according to the International Classification of Diseases, Tenth Revision, (ICD-10) criteria. The participants were assessed using a self-report questionnaire. The parameters of assessment were aggressive behavior, domestic violence, workplace violence, verbal abuse, and variables including the forensic aspects of alcohol consumption, such as road traffic accidents, etc. Previous research and similar studies on factors related to alcohol dependence syndrome were compared to establish a conclusion for the study.

Results

Participants reported to have decreased psychomotor function upon alcohol consumption compared to the time they were not under the influence of alcohol. Aggressive behavior associated with irritability and agitation was observed in 89.28% (50 out of 56) participants. A total of 76.78% (43 out of 56) had road traffic accidents at least once under the influence of alcohol. Of the sample, 85.71% (48 out of 56) committed verbal abuse at the workplace and home as a result of aggression under the influence of alcohol. And 69.64% (39 out of 56) of the sample had memory loss after consumption of alcohol.

Conclusion

There are several behavioral changes in individuals who are alcohol dependent, which may affect their day-to-day activities and cause poor performance in the workplace. Participants in the study showed a notable positive relation between alcohol dependence syndrome and aggressive behavior, verbal aggression, domestic violence, memory loss, and road traffic accidents under the influence of alcohol. Alcohol dependence syndrome can be linked with decreased quality of life due to problems faced in daily activities like psychomotor functions, sleeping, etc. During the treatment of alcohol dependence or withdrawal from alcohol, individuals experience socio-behavioral changes. Cognitive behavior therapy, including cognitive neuroscience, can help in managing these behavior and personality changes in alcohol dependence syndrome.

## Introduction

Alcohol dependence is described as a collection of behavioral, cognitive, and physical symptoms that emerge after repeated alcohol consumption. These symptoms often involve an intense craving for alcohol, challenges in regulating its consumption, continuing to use it despite adverse consequences, prioritizing alcohol over other responsibilities, developing a tolerance, and, in some cases, experiencing physical withdrawal [1]. Alcohol possession, consumption, and use have been common for many years due to its versatility. Stoppage or reduction of alcohol consumption in alcohol dependence syndrome leads to alcohol withdrawal [2]. The statistics of studies in Indian setting show the prevalence of alcohol consumption over the years, such as 23.7% in 2010, Bangalore, 28.78% in 2016, Kerala, 35% in 2017, Andaman and Nicobar Islands, 38.2% in 2018, rural Indore, and 44% in 2019, Dehradun [1]. Alcohol consumption has various physical and psychological effects, such as obesity, liver disorders, anxiety, and suspicion.

Higher alcohol intake might be a way for individuals to deal with challenging emotions or situations stemming from their exposure to violent incidents at work [3]. Over-consumption of alcohol to avoid stress might lead to alcohol addiction [4]. Most men who are lifetime pathological drinkers have their first drink at a younger age of 21-25 years [5]. Rehabilitation centers are focused on promoting abstinence and preventing further harm. During the withdrawal of alcohol, alcohol abstinence self-efficacy affects the subjective well-being of the alcohol drinkers undergoing treatment for alcohol dependence syndrome [6]. It has been observed that there were changes in the social well-being and demeanor of the individuals during the withdrawal of alcohol [7]. Aggressive behavior is a standard behavioral change in alcohol abuse patients [8]. Alcohol-related violence can victimize both spouses and children, and children may also experience health and social issues that could persist into adulthood [9]. A study conducted at Madras Medical College, Chennai, India, has found a positive association between alcohol dependence and biological and behavioral markers of impulsive behavior and aggression [10]. In a survey on intoxicating aggression, a statistically significant rise in aggression has been shown at doses of 0.75 g/kg and above [11]. A study showed that a considerable proportion of injured (2 to 33 percent) or killed (6 to 48 percent) road users in India had consumed alcohol before the accident [12].

Antisocial personality disorders are generally more frequently observed in men, whereas affective and anxiety disorders tend to be common vulnerability factors in women [13]. Family history was less prevalent than antisocial personality history for the course of alcoholism in another study [14]. The environment, family, peers, and adversities influence the initiation and progression of alcohol abuse [15]. Other studies conclude that males of average age 35 years were primarily physical and emotional abusers and aggressive in nature. However, there was no relation between alcohol abuse and physical abuse among older adults [16].

Bombay Prohibition Act of 1949 proscribed the sale, purchase, and consumption of alcoholic drinks [17]. The Motor Vehicles Act of 1988 makes it illegal to drive while under the influence of alcohol or drugs. It specifies that having a blood alcohol level of 30 mg per 100 ml of blood or higher is considered a legal offense [1,18]. Long-term and excessive alcohol consumption raises the risk of dementia due to alcohol's harmful effects on the brain and the nutritional deficiencies that can result from it. Older individuals with cognitive problems are more likely to break these legal rules if they have substance abuse issues [19]. Young men are primarily involved in road traffic accidents during the night and on weekends under the influence of alcohol above the statutory limit (30 mg%) [20]. Laborers are the victims of road traffic accidents and being knocked down [21].

Based on a review, alcohol can be attributed as a contributing factor to domestic violence [22]. Some studies demonstrated that alcohol use was associated with an increased possibility of physical injury [23]. Increasing taxes on liquor/beer have proved to be effective in reducing violence, while restrictions on advertising and increase in illegal drug price have negligible effects [24]. It also leads to a reduction in work performance. One of the studies linked the consumption of alcohol to work performance in individuals working at an office. Individuals who consumed alcohol were doing less work, leaving early, and being late to work [9]. Alcohol may also play a role in unintentional injuries. A study concluded that older workers having two drinks per day had fewer injuries than those with five or more drinks per day [25]. Most alcohol-related offenses are crimes of violence, such as aggravated assault and homicide [26]. In addition to the harm that alcohol consumption causes to the alcohol drinkers themselves, it may have a broader impact on family members, especially spouses and children, which has social consequences [27].

Alcohol dependence leads to irritable and aggressive behavior in individuals. It also leads to loss of appetite, sweating, anxiousness, sleep disturbances, hallucinations, suspicion, changes in mood, pacing nature, and blackouts [28-31]. During the treatment of alcohol addiction or alcohol withdrawal, individuals experience socio-behavioral changes. Assessing these behavioral and personality changes in patients during the treatment of alcohol dependence syndrome helped in managing the patients and learning about its impact on the forensic history of the patient. Coping strategies are suggested after the assessment of the behavioral and personality changes in alcohol dependence syndrome to understand the socio-behavioral pattern and to aid in better management of the patient.

The objectives of the study are to assess the behavioral and personality changes in alcohol dependence syndrome in Wardha, Maharashtra, Central India, to assess the forensic aspects of alcohol dependence syndrome in Wardha, and to compare the data collected with existing research or previous studies. It was observed that there was a rise in admission cases of alcohol-dependent individuals post-COVID-19 lockdown, with a spike from 14% to 27% in a study conducted in Wardha [2]. In a survey conducted in Wardha, there was a decline in alcohol consumption post-covid-19 lockdown, which led to a rise in cases of alcohol withdrawal [2]. Alcohol withdrawal in individuals occurs as a result of alcohol dependence syndrome. In our study, we focus on behavior and personality changes in alcohol dependence syndrome.

## Materials and methods

The study is an observational study. The study was conducted after receiving the approval of the Institutional Ethics Committee Datta Meghe Institute of Medical Sciences with the reference number DMIMS(DU)/IEC/2022/286. The confidentiality of the participants was maintained throughout the research. An informed consent was signed before the participation of individuals in the study. The sample size for the study was found using the Morgagni rule. Attributable to the various laws imposed in Wardha regarding the consumption and limited use of alcohol, the study population is smaller in sample size. The study was conducted at a rural tertiary care hospital, Acharya Vinoba Bhave Rural Hospital, Wardha, Maharashtra, Central India. The participants were alcohol-dependent individuals from neighboring regions of Wardha seeking treatment in a rural tertiary care hospital.

The selection criteria for the study can be described as follows. The study included participants undergoing treatment for alcohol dependence syndrome, patients being treated for withdrawal of alcohol, participants who had a history of chronic alcohol use, participants who could be available for the in-person interview, and participants who were willing to participate in the study. The study excluded individuals who were not willing to participate in the study, individuals who were in the critical care unit due to alcohol withdrawal, and individuals not oriented to time, place, and person.

A total of 62 individuals participated in the study. Out of 62 participants, six were excluded as they were uncooperative in providing data. A total of 56 participants were included in the study. All the participants involved in the study were males. The responses were recorded for every participant individually at a rural tertiary care hospital in a self-reported questionnaire for assessment of behavioral and personality changes in alcohol-dependent individuals.

The study was divided into four phases. In the first phase, the participants were informed of the behavioral and personality changes that occur in alcohol dependence syndrome. In the second phase, the participants were asked to give their responses to the Cut-Down, Annoyed, Guilty, and Eye-Opener (CAGE) questionnaire and the Alcohol Use Disorders Identification Test (AUDIT) questionnaire. The diagnosis of alcohol dependence syndrome was made according to the International Classification of Diseases, Version 10, (ICD-10) criteria. The ICD-10 criteria helped in differentiating alcohol dependence syndrome from other psychiatric disorders. In the third phase, the data was collected based on the parameters of assessment of behavioral and personality changes in alcohol dependence syndrome. The forensic aspects of alcohol dependence syndrome were included in the parameters of the evaluation. Descriptive statistics have been used for statistical analysis. The data was analyzed on Microsoft Excel 2019 (Microsoft Corporation, Redmond, Washington, United States). In the fourth or final phase, the data derived from the study was compared with existing research conducted on alcohol dependence syndrome. All the discrepancies in the input and analysis of data were removed. The validity of responses in the interview during the study was crosschecked by the relatives, and records of history were taken during the participants' stay at the hospital. The study was conducted for a duration of six months, from January 2023 to June 2023. The primary outcome measure is the behavioral and personality changes in alcohol dependence syndrome.

Parameters of assessment of behavioral and personality changes in alcohol dependence syndrome

The participants were enquired about factors involving the physical, behavioral, and psychological aspects. The participants were majorly assessed on the forensic aspects due to their alcohol dependence. The selected participants were evaluated based on parameters such as aggressive behavior, domestic violence, violence at work, verbal abuse, memory loss, suspiciousness, road traffic accidents, legal charges, and problems faced in day-to-day activities. Components of forensic psychiatry have been included in the parameters of assessment of alcohol dependence syndrome.

## Results

Sociodemographic data of participants

There were a total of 62 participants. Out of which, 100% were male participants. There were no females admitted for alcohol dependence syndrome during the study period. A total of 30.64% were farmers, 27.4% were daily-wage laborers, 22.58% were unemployed, 12.9% had corporate jobs, and 6.45% were businessmen. The majority of the participants were in the age range of 40-50 years, constituting 53.22% of the total participants. A total of 62.9% of participants were from the urban population, and 37.09% were from the rural population. A total of 56.45% of participants belonged to the middle-class socioeconomic background, while 43.54% belonged to the lower-class socioeconomic background according to the modified BG Prasad socioeconomic scale for India. Most of the participants reported to have started alcohol consumption under peer pressure. The following table depicts the sociodemographic data of the alcohol-dependent individuals admitted to the hospital (Table 1).

**Table 1:** Sociodemographic data of alcohol-dependent individuals

Factors		Number of participants (Total= 62)	Percentage (%)
Gender	Male	62	100%
Occupation	Farmer	19	30.64%
	Daily-wage laborer	17	27.41%
	Businessman	4	6.45%
	Corporate job	8	12.9%
	Unemployed	14	22.58%
Age	18-28 years	5	8.06%
	29-39 years	14	22.58%
	40-50 years	33	53.22%
	51-61 years	10	16.12%
Area of residence	Rural	23	37.09%
	Urban	39	62.9%
Socioeconomic status	Low	27	43.54%
	Middle	35	56.45%
	High	0	0%

Behavior and personality changes in alcohol dependence syndrome

Out of 62 participants, six were uncooperative while providing the forensic history during the study. The data was recorded for 56 participants. A total of 89.28% of the participants showed aggressive behavior and 87.5% faced problems in carrying out day-to-day activities such as household chores, sleeping, decision-making, eating, laundry, etc. A total of 85.71% of participants had engaged in verbal abuse at the workplace and home, 78.57% of participants had engaged in domestic violence, 76.78% had been in road traffic accident, 69.64% had memory loss, 28.57% had engaged in violence at work, and 7.14% had suspiciousness regarding their partner or family members. A total of 7.14% of participants had legal charges against them due to offenses committed under the influence of alcohol. The following table depicts the behavior and personality changes, including the components of forensic psychiatry, in alcohol dependence syndrome (Table 2).

**Table 2:** Behavior and personality changes including the forensic aspects of alcohol dependence syndrome

Variables	Number (out of 56)	Percentage (%)
Aggressive behavior	50	89.28%
Domestic violence	44	78.57%
Violence at work	16	28.57%
Verbal abuse	48	85.71%
Memory loss	39	69.64%
Suspiciousness	4	7.14%
Road traffic accident	43	76.78%
Police history/legal charges/conviction	4	7.14%
Problems faced in carrying out day-to-day activities	49	87.5%

Aggressive behavior associated with irritability and agitation was observed in 89.28% of participants. The remaining participants (10.72%) reported that they relaxed after consuming alcohol and were slow in processing things. Verbal abuse and domestic violence were common in alcohol-dependent individuals. It was observed that domestic violence led to an increased number of cases of dysfunctional families. It also affected the psychological and behavioral characteristics of the alcohol-dependent participants. Participants admitted for road traffic accidents had a motorbike crash or lost control of the vehicle. Legal charges or convictions were of a low ratio (0.071). Few participants had first information report (FIR) charges against them due to legal offenses. It was observed that participants with legal charges against them had psychiatric disorders associated with alcohol dependence syndrome.

Frequency of some notable forensic aspects in alcohol dependence syndrome

The total number of participants who knew how to ride vehicles was 51, out of which 43 had road traffic accidents at least once. In total, 60.78% of participants had a road traffic accident once, 13.72% had a road traffic accident twice, and 9.80% had a road traffic accident thrice under the influence of alcohol, whereas 15.68% never had a road traffic accident under the influence of alcohol. The following tables were prepared in accordance with the frequency of the aspects observed in alcohol-dependent individuals (Table 3).

**Table 3:** A history of road traffic accidents under the influence of alcohol

Instances of road traffic accidents under the influence of alcohol	Number of participants (out of 51)	Percentage (%)
Once	31	60.78%
Twice	7	13.72%
Thrice	5	9.80%
Never	8	15.68%

The total number of participants who had a history of domestic violence as cross-checked with relatives was 44. A total of 52.27% (23 out of 44) of participants committed domestic violence almost daily, 6.81% committed domestic violence at least once a week but not daily, and 40.90% committed domestic violence once or twice a month. The following table depicts the data for acts of domestic violence or inflicting physical harm to the family under the influence of alcohol (Table 4).

**Table 4:** A history of domestic violence under the influence of alcohol

Frequency of domestic violence under the influence of alcohol	Number of participants (out of 44)	Percentage (%)
Almost daily	23	52.27%
At least once a week but not daily	3	06.81%
Rarely (once or twice a month)	18	40.90%

The following table depicts the data on verbal abuse engaged in by the participants under the influence of alcohol. Here, "frequently" refers to acts of verbal abuse done almost daily, "occasionally" refers to at least once a week but not daily, and "rarely" refers to once or twice a month. A total of 48 participants had done an act of verbal abuse at least once under the influence of alcohol. In total, 45.83% had engaged in verbal abuse almost daily or frequently, 39.58% had engaged in verbal abuse occasionally but not daily, and 14.58% had done acts of verbal abuse on rare occasions (Table 5).

**Table 5:** A history of verbal abuse under the influence of alcohol

Acts of verbal abuse under the influence of alcohol	Number of participants (out of 48)	Percentage (%)
Frequently	22	45.83%
Occasionally	19	39.58%
Rarely	7	14.58%

The following table depicts the data of the first information report (FIR) or police involvement for offenses committed under the influence of alcohol. A total of four participants had legal charges against them. Out of these, 50% had more than three legal charges against them, 25% had two legal charges against them, and 25% had one legal charge against them. It shows us the prevalence of criminal acts and the effect of alcohol on criminal offenses (Table 6).

**Table 6:** Police history/legal charges stemming from the influence of alcohol

Instances of police history/ FIR charges/legal charges under the influence of alcohol	Number of participants (out of 4)	Percentage (%)
More than three	2	50%
Two	1	25%
One	1	25%

Most participants reported being unable to work without the first drink in the morning. It was a cause for the feeling of anxiousness and continuous drinking process. Most of the participants also reported to have liver dysfunction and were being treated for liver disorders. Participants reported that they usually drank in groups or with their peers, which led to their uncontrollable drinking patterns.

Distribution of alcohol-dependent case admissions during the study period

It was observed that there was an influx of admissions (30.64%) of alcohol-dependent patients after festivals in India in March. It was 8.06% in January and 11.29% in February. There were 14.51%, 16.12%, and 19.35% admissions in May, June, and April, respectively (Table 7).

**Table 7:** Distribution of the number of admissions of alcohol-dependent individuals in the rural tertiary care hospital, Wardha (month-wise)

Month of admission (Year 2023)	Number of admissions (Total=62)	Percentage (%)
January	5	8.06%
February	7	11.29%
March	19	30.64%
April	12	19.35%
May	9	14.51%
June	10	16.12%

Family history of consumption of alcohol

Out of 62 total participants, 34 participants had a family history of alcohol consumption. They reported that their father or a close relative, like the paternal or maternal uncle, consumed alcohol regularly. This data could be linked to the genetic association of alcohol dependence syndrome. A total of 54.83% of participants had a family history of alcohol consumption. All the male participants in the study were willing to take measures to reduce their alcohol consumption.

## Discussion

Comparison of results with previous studies

In previous studies, aggression-hostility, sensation seeking, socialization, expressiveness, suspicion, and mistrust were the personality traits that were linked to alcohol dependence syndrome [32,33]. Substantial aggressive behavior was noted in our study on alcohol-dependent participants, with about 89.28% eliciting aggression on trivial matters under the influence of alcohol, which did not agitate them usually. A total of 7.14% of participants suspected their partners or family members of being untrustworthy. They showed mistrust and suspicion over small matters under the influence of alcohol, as reported by their family members. It depicts a positive relation of a suspicious nature due to the consumption of alcohol in a small proportion of the sample.

Physical violence at work and home is common in alcohol dependence syndrome, as observed in previous studies [9]. A study showed a positive association between workplace aggression, including physical and verbal aggression, in alcohol-dependent individuals [34]. Our findings show that 85.71% of participants had engaged in verbal abuse at the workplace and home under the influence of alcohol. Further statistics show verbal aggression toward their colleagues was common after the consumption of alcohol. A total of 45.83% (22 out of 48) participants had engaged in verbal abuse almost daily or frequently, and 39.58% (19 out of 48) participants had engaged in verbal abuse occasionally but not daily. The physical violence at the workplace was a result of aggravation of verbal aggression that was committed by 28.57% (16 out of 56) participants. Various studies have estimated that up to 50% of alcohol-dependent men display violent behavior [35]. In our study, 78.57% (44 out of 56) of males showed a positive relation of domestic violence with the consumption of alcohol. Aggressive behavior is promoted by the cognitive impairments that can result from both short-term and chronic alcohol use [35]. A total of 52.27% (23 out of 44) of males committed domestic violence almost daily. This behavior may have occurred as a result of provocation in the beginning for most alcohol-dependent individuals, while it led to the formation of the habit of domestic violence. A total of 40.90% (18 out of 44) of males who committed domestic violence once or twice a month are at a chance of being daily offenders of domestic violence. A lower ability to handle stress can strengthen the tendency to act aggressively in a situation when influenced by alcohol [35]. Heavy consumption of alcohol in a short duration is more suggestive of promoting aggressive behavior in individuals than chronic use of alcohol [36,37]. The anticipation of the outcome associated with alcohol consumption, known as alcohol outcome expectancies, plays an essential role in verbal abuse and physical aggression. The memory cells of alcohol outcome expectancies are activated when alcohol is consumed [35].

Genetic association of alcohol dependence can be implied based on data collected in the study. In a previous study, individuals with a family history of alcohol consumption were found to be at a higher risk of developing the disorder compared to those without such a familial background [38]. In our findings, 54.83% (34 out of 62) participants had a positive family history of alcohol consumption. Positive family history can be positively linked with the incidence of alcohol dependence in the participants. Further research can be done on the age of first drink and alcohol dependence to determine the risk of alcohol dependence in individuals with a positive family history of alcohol consumption. Heavy alcohol consumption leads to an increased likelihood of cirrhosis and certain cancers [39]. A previous study summarized that 50% of the deaths resulted from liver cirrhosis, 22% were due to interpersonal violence, 22% were linked to self-harm, 15% were a result of traffic accidents, and 12% were associated with liver cancer, all of which were attributed to alcohol consumption [1]. The participants of our study were being treated for liver disorders like liver cirrhosis and thiamine deficiency. Early intervention and prevention of alcohol dependence can prevent such deadly injuries and lead to the reversal of some fatal diseases. A study in India reports that 14.9% of road traffic accident victims were found to have consumed alcohol [21]. Our findings show that 76.78% (43 out of 56) participants had road traffic accidents. It must be due to psychomotor dysfunction and muscular incoordination under the influence of alcohol. Our sample findings in Wardha showed that 60.78% (31 out of 51) had motorbike crashes or road traffic accidents once, which gives notable positive results on the traffic injuries caused by alcohol consumption. In the study conducted in Khammam, 21% of the study participants experienced road accidents. Additionally, 15% of the sample engaged in criminal activities and violence, 7.5% displayed self-harming behavior, and 16.5% were involved in risky sexual behaviors [40]. Our findings show that alcohol dependence can be positively linked with high-risk behavior, such as physical violence at work, domestic violence, aggressive behavior, crime, and road traffic accidents.

International surveys show higher rates of drinking in metropolitan areas or urban areas [41]. In our findings, we noted that 62.9% (39 out of 62) participants were from the urban population, which shows that the prevalence of alcohol consumption is higher in urban areas than in rural areas. Alcohol dependence syndrome was observed more in the population of 40-50 years of age in our study. The stressful environment of the workplace may pose a risk for alcohol dependence in individuals [42]. It is observed more in laborers such as farmers (30.64%) and daily-wage laborers (27.41%) and less in unemployed participants (22.58%). It could be due to work-related stress and strenuous field work, which did not yield much output in relation to the input given in labor. It was seen more in the participants from middle socioeconomic backgrounds than from low socioeconomic backgrounds. It might be due to the monetary stress and failure to meet expectations in a middle-class household. The increased number of admissions of alcohol dependence syndrome patients in March must be due to the increased consumption of alcohol due to the habit of drinking owing to the festivities.

Cognitive impairment in alcohol dependence syndrome

In the study, memory loss was experienced by 69.64% (39 out of 56) participants with alcohol dependence syndrome. In comparison, 87.5% (49 out of 56) reported having problems carrying out daily activities such as household chores, sleeping, decision-making, etc. Alcohol dependence occurs as a result of alcohol abuse during social drinking. It is characterized by the brain reward and stress symptoms. Altered brain function can lead to excessive and uncontrollable drinking by alcohol-dependent individuals. It can manifest as alcohol withdrawal once the consumption of alcohol is stopped or reduced. It leads to motivational and re-engaging in excessive drinking behavior on abstinence. It leads to relapse and a negative emotional state during abstinence from alcohol [28]. Alcoholism is a pertinent problem marked by recurrent relapses, uncontrollable drinking, and difficulties in social and work-related functioning [43]. It occurs as an allostatic mechanism by activating brain circuits involved in compulsive behavior, such as the cortico-striatal-thalamic loop [44]. Most often, alcohol-dependent individuals have memory loss after drinking alcohol and deviate from giving actual information [45]. The harmful effects of long-term alcohol use on brain signaling, synaptic communication, and cellular damage can result in primary alcoholic memory loss or dementia [46]. Alcohol dependence also raises the likelihood of sustaining injuries, which may be attributed to alcohol-related issues such as reduced coordination and balance, longer reaction times, and impaired attention, perception, and judgment [47].

Alcohol dependence leads to various cognitive impairments, such as memory and psychomotor impairments, such as pacing, tapping toes or fingers, restlessness, etc. Recovery from these impairments depends on the length of abstinence from alcohol [48]. Studies have shown that alcohol dependence leads to impaired decision-making and poor working memory processes [49]. The gamma-aminobutyric acid (GABA)-A neuron is an inhibitory neuron that has a crucial function in regulating neural circuits and cognitive processes [50]. Specific GABA-A receptor subunits could potentially influence the genetic predisposition to alcohol dependence [51]. Excessive alcohol consumption seems to lead to cognitive deficits in the cortex. It causes mild to moderate memory impairments due to a combination of direct neurotoxic effects and pre-existing risk factors [52]. Drinking alcohol produces an altered state, which reinforces the properties of alcohol and its impact on the brain in chronic alcohol use. It leads to a loss of conscious mind for decision-making, problem-solving, and planning behavior [53]. Our findings showed legal charges against 7.14% of the participants. Cognitive impairment and absence of a conscious state of mind lead to violation of laws and offenses committed under the influence of alcohol.

Treatment and strategies to manage alcohol dependence syndrome

The drug disulfiram blocks and hinders the activity of the enzyme aldehyde dehydrogenase. This inhibition results in an increased level of acetaldehyde in the body when alcohol is consumed and triggers symptoms like flushing, shortness of breath, rapid heartbeat, headaches, and nausea. Disulfiram is used to encourage patients to abstain from alcohol consumption [54]. To prevent alcohol dependency, the relatives of the patients should be well-informed and aware of the situation. Alcohol-dependent patients should seek medical help with the encouragement of family members. Most participants have reported that they have tried to stop the consumption of alcohol but to no use with their habit aggravating due to their family matters or financial condition. The use of cognitive neuroscience in psychosocial treatment can be helpful for alcohol dependence syndrome. The relapse of alcohol abuse is an acquired behavior that can be controlled using cognitive behavioral techniques in addition to pharmacological treatment, as seen in a study conducted in Madras Medical College, India [10].

Imposing taxes may not be an effective step in curbing the consumption of alcohol. The laws in India are breached regularly as alcohol consumers easily get access to illicit liquor and substances. Poor people may turn to illegal methods of alcohol production or brewing and may become a victim of methanol poisoning [1]. Alcohol dependence can be managed by deviation from the root cause of alcohol consumption. Stress increases cortisol levels in the body. Cortisol can also impact an individual's cognitive functions by promoting habit-based learning, potentially contributing to the formation of habits and an increased risk of relapse [38]. Alcohol-dependent individuals should be encouraged to practice other activities that help them manage stress and avoid the thought of alcohol consumption. Family members should support alcohol-dependent individuals in managing their stress and help them reduce their consumption of alcohol. Ultimately, it depends on the self-will and motivation of alcohol-dependent individuals to curb their consumption of alcohol.

Limitations of the study

Most participants were conscious and cooperative during the study. However, the information given by the participants and the relatives did not match at times; there was a need to rely on the history records of the hospital for the participants. Some participants could not recollect some information during the history-taking process. There may have been a bias in self-reported data since the information given by participants and their relatives was noted as incongruent. There may have been under-reporting or over-reporting due to the complexity of questions, lack of understanding of the questionnaire, and false responses. There may be a bias based on comorbidities such as liver cirrhosis, thiamine deficiency, or concurrent psychiatric disorders like schizophrenia, bipolar disorder, and attention deficit hyperactivity disorder.

## Conclusions

Alcohol contributes to the aggressive behavior of individuals. It also suggests an increase in the risk of road traffic accidents. The quality of life of alcoholic individuals is hampered due to the high-risk behaviors under the influence of alcohol. Laws prohibiting drinking and driving have curbed road traffic accidents. However, alcohol takes away the ability of an individual to carry out conscious action, which is required to process information and for logical thinking. The study suggests a positive association between alcohol dependence and road traffic accidents under the influence of alcohol. Domestic violence and aggressive behavior under the influence of alcohol can be positively related to alcohol dependence syndrome. Cognitive deficits, including impaired decision-making and memory loss, are associated with alcohol dependence.

The research also indicates a positive relation between workplace aggression like violence, verbal abuse, and alcohol dependence. Suspiciousness and hostility can be related to alcohol dependence syndrome. Genetic factors are implicated, with individuals having a family history of alcohol consumption being more likely to develop alcohol dependence. The study also points out a high incidence of road traffic accidents among alcohol-dependent participants, likely due to psychomotor dysfunction and muscular incoordination while intoxicated. The study underscores the prevalence of alcohol consumption in urban areas and its association with high-risk behaviors like aggression, domestic violence, and crime. The study notes that alcohol dependence is more common among individuals in their 40s-50s and those in stressful work environments, such as farmers and daily-wage laborers. Imposing taxes on liquor may not help in curbing the excessive alcohol consumption. The use of disulfiram, family support, and cognitive behavioral therapies, including cognitive neuroscience, can help in the management of alcohol dependence syndrome. Stress should be managed, and deviation from the primary cause of alcohol consumption should be encouraged among alcohol-dependent individuals. Further studies are required on the effects of cognitive behavior therapies on alcohol dependence syndrome. More studies are needed on the pharmacotherapy and impact of the drugs on the brain pathophysiology of alcohol-dependent individuals.
